# First DNA barcode reference library of grasshoppers on the Comoros Archipelago

**DOI:** 10.3897/BDJ.14.e188335

**Published:** 2026-05-14

**Authors:** Pauline Alwine Wasle, Oliver Hawlitschek, Sylvain Hugel

**Affiliations:** 1 Faculdade de Ciências e Technologia, Faro, Portugal Faculdade de Ciências e Technologia Faro Portugal; 2 University of Zurich, Zurich, Switzerland University of Zurich Zurich Switzerland; 3 Institut des Neurosciences Cellulaires et Intégratives, Strasbourg, France Institut des Neurosciences Cellulaires et Intégratives Strasbourg France

**Keywords:** grasshoppers, Comoros, COI, DNA barcoding, species identification

## Abstract

**Background:**

DNA barcoding studies utilising COI gene sequences have been widely applied in insect taxonomy for two decades and remain so. Barcode reference libraries are a valuable molecular resource for future biodiversity assessments, including standard barcoding surveys and environmental DNA (eDNA) approaches. Grasshoppers, members of the suborder Caelifera of the insect order Orthoptera, on the Comoros Islands have not yet been subject of barcoding studies, marking a clear gap in a poorly-studied region of high biodiversity and endemism. Our results illustrate both the potential and the constraints of CO1 barcodes in caeliferan systematics and highlight the importance of expanding molecular reference libraries in understudied biodiversity hotspots.

**New information:**

We provide a first barcode reference library, including COI sequences from 22 Caelifera specimens representing 12 genera and 15 species. Nine out of 12 genera can be reliably identified using DNA barcodes. However, closely-related taxa, such as *Symbellia* species, were not well resolved by CO1 alone, highlighting the need for additional markers and morphological evidence. Furthermore, preliminary evidence suggests the presence of different *Parepistaurus* lineages on the Archipelago, potentially indicating undescribed diversity and deserving further study.

## Introduction

The Comoros Archipelago, located in the Western Indian Ocean between the African continent and Madagascar, comprises four main islands: Grande Comoro, Mohéli, Anjouan and Mayotte. All islands were formed through volcanic activity and were never connected to any other landmasses (Harris and Rocha 2009), which classifies them as true oceanic islands (Weyeneth et al. 2008). Historically, the islands were noted for their relatively undisturbed, continuous forest cover and low population density, yet, in recent decades, they have experienced significant deforestation due to anthropogenic pressures (Goodman et al. 2010). At least 80% of the native vegetation has been destroyed since human colonisation and population numbers continue rising steadily (Harris and Rocha 2009).

The Comoros are characterised by tropical climate and vertical zonation of habitats fostering high species richness and endemism (Agnarsson and Kuntner 2012). Most terrestrial animal lineages of Comoros trace their origins to colonists from Madagascar, with a smaller proportion arriving from mainland eastern Africa (Weyeneth et al. 2008, Rocha et al. 2010). Certain taxa, especially insects, also show links to Southeast Asia (Weyeneth et al. 2008). After colonisation, some lineages considered native maintained genetic exchange with their source populations. In the isolated ecosystems of the Comoros Islands, other species became cut off from their source populations and evolved into endemic species, in some cases undergoing subsequent cladogenesis and radiations that generated the present-day diversity. For Orthoptera, it is, for example, the case for crickets of the genus *Ornebius*, which most likely originated from Southeast Asia and subsequently radiated across the islands of the south-western Indian Ocean, including the Comoros (Warren et al. 2016).

Grasshoppers (Orthoptera, Caelifera) play vital roles in local ecosystems as herbivores and prey (Strickland et al. 2013). While the knowledge of orthopterans on Madagascar improved significantly (e.g. Hugel (2023)), still little is known about grasshopper diversity on the Comoros. Difficulties encountered during field observations and morphological examination of specimens include: (1) cryptic species that are morphologically conservative; (2) phenotypic variability within the same species and (3) insufficient species-specific features in juvenile and sub-adult specimens (Hansen et al. 2017).

These problems have been addressed in recent years using molecular techniques like DNA barcoding (Hebert and Gregory 2005, Song 2010). In animals, the mitochondrial cytochrome c oxidase I (COI) gene has been established as the standard barcoding marker (Hebert et al. 2003). So far, millions of COI barcode sequences have been collected and uploaded to the *Barcode of Life Database* (BOLD; Ratnasingham and Hebert (2007)) and used in taxonomic studies (Trivedi et al. 2016, Hawlitschek et al. 2017). One main advantage of the barcoding approach is its applicability to organisms with very large genomes, in which sequencing the entire genome is very costly and not always feasible. Orthopterans commonly have genomes as large as 6 GB, in extreme cases up to 22 GB (Hawlitschek et al. 2023), making this molecular tool especially useful.

This study employs DNA barcoding to investigate the genetic diversity of caeliferan grasshoppers from the Comoros Islands, based on COI sequences generated from specimens collected across the Archipelago. Rather than aiming to establish a comprehensive or updated species checklist, we focus on evaluating the effectiveness of COI barcodes for species delimitation within this group, drawing attention to both their utility and limitations, particularly in taxa such as *Symbellia*, where resolution is poor. The barcode reference library presented here provides a valuable molecular resource for future biodiversity assessments, including standard barcoding surveys and environmental DNA (eDNA) approaches. In particular, it offers a foundation for investigating the diet of insectivorous vertebrates, endemic to the region through metabarcoding of faecal or regurgitated material. Furthermore, the dataset reveals preliminary signals of potentially undescribed or cryptic diversity, notably within *Parepistaurus*, underscoring the need for further integrative taxonomic work.

## Materials and methods

### Sampling and species identification

**Figure 1.**
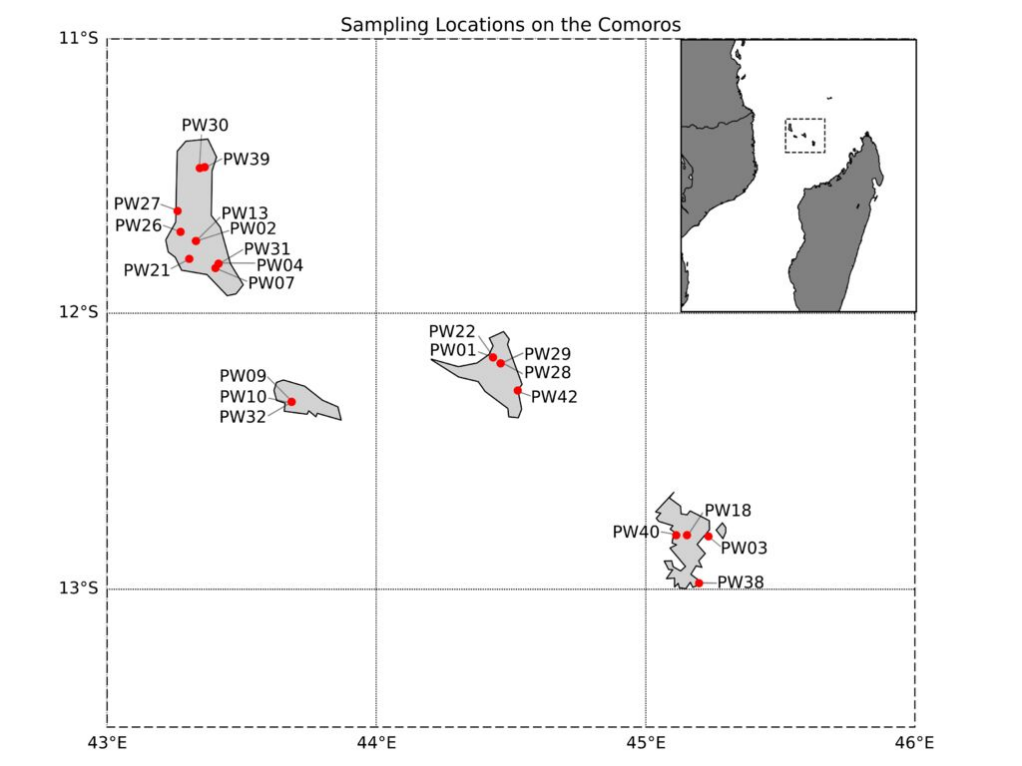
Sampling locations of sequenced orthopteran individuals marked in red. Islands from left to right: Grand Comoro, Mohéli, Anjouan and Mayotte.

Specimens were collected by Sylvain Hugel from various locations across the Comoros Archipelago (Fig. 1) between 2010 and 2024. Insect collection was conducted under the appropriate authorisations granted by the Centre National de Documentation et de Recherche Scientifique and the Musée National des Comores (CNDRS/39/10, CNDRS/082/10, CNDRS/083/10, CNDRS/40/10), as well as by the Office National des Forêts (ONF) and the Direction de l’Environnement, de l’Aménagement et du Logement (DEAL) of Mayotte. In this article, 'Comoros' is used as a geographic term, without reference to political status. It refers to the Comoros Archipelago as a whole, encompassing the islands of Grande Comoro, Mohéli, Anjouan and Mayotte.

Morphological species identification was initially conducted by Sylvain Hugel, using literature and identification keys specific to the Comoros Islands and the Western Indian Ocean region. Key references included works by Chopard (1958), Rehn (1959) , Dirsh and Descamps (1968) and Descamps and Wintrebert (1969). Holotypes of relevant species deposited in the Muséum national d’Histoire naturelle (MNHN) were also examined. Each specimen was either entirely preserved dry or had one leg immediately placed in 100% ethanol (EtOH), while the remainder of the specimen was kept dry.

### Sample processing

We performed DNA extractions from muscular tissue of grasshopper femora of 42 individuals. The femora were cut open lengthwise and muscle tissue was removed for further processing. For small specimens, the entire femora were used. The DNA was extracted using the Qiagen DNeasy Blood & Tissue Kit (Qiagen, Germany), following the manufacturer’s protocol. DNA quantity and quality were assessed using a Nanodrop spectrophotometer and verified by agarose gel electrophoresis. The standard barcoding region of the mitochondrial gene, COI, was then amplified in a PCR using the primer pair COBL (forward, 5' TYTCAACAAAYCAYAARGATATTGG 3') and COBU (reverse, 5' TAAACTTCWGGRTGWCCAAARAATCA 3'). These primers are specifically designed for amplification of orthopteran DNA (Huang et al. 2013). The PCR reactions were performed in a 50 μl reaction volume. The thermal cycling conditions for amplification were as follows: initial denaturation at 94°C for 3 min; 34 cycles of denaturation at 94°C for 30 s, annealing at 49°C for 30 s and extension at 72°C for 90 s; and a final extension step at 72°C for 8 min. PCR products were then checked for amplification on an agarose gel and successful samples were purified using a QIAquick PCR Purification Kit. Purified products were sequenced using the Sanger sequencing method by an external provider (Microsynth, Balgach, Switzerland).

### Data processing and analyses

**Figure 2.**
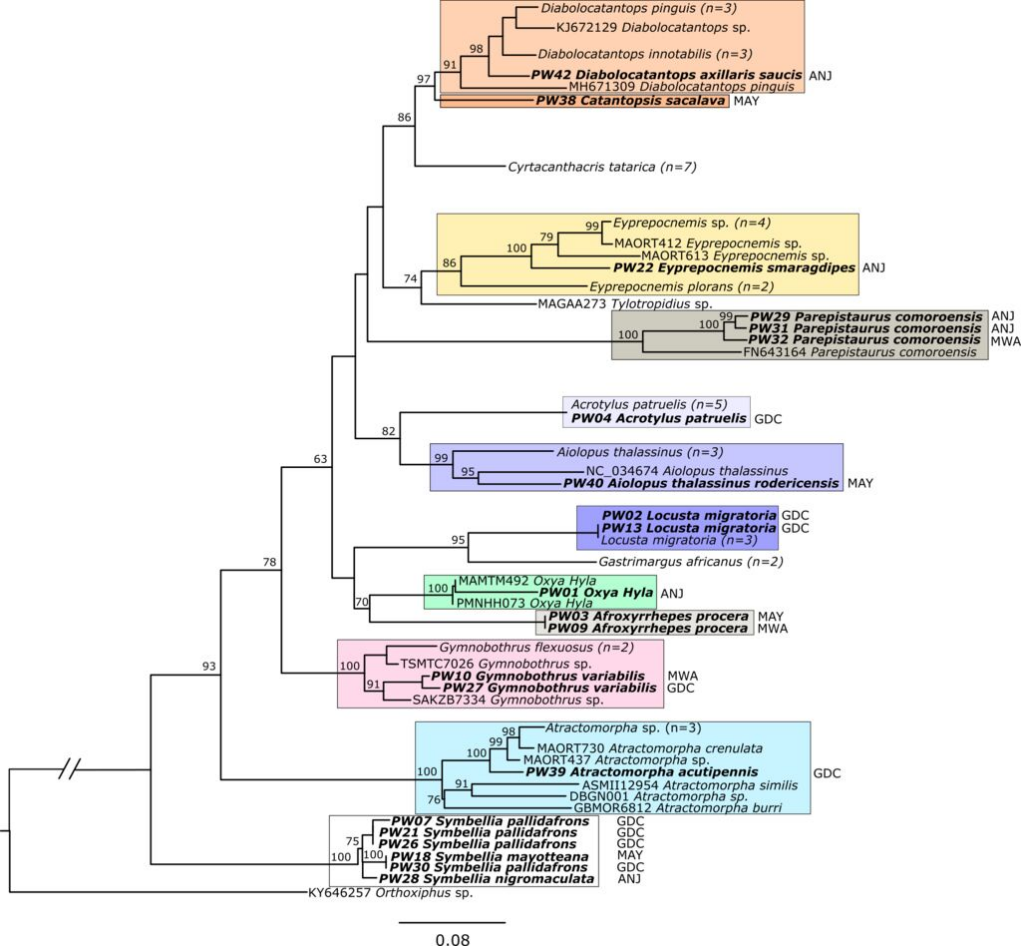
Samples of orthopteran individuals generated in this study are bold and clades belonging to the same subfamily are highlighted in different shades of the same colour. The 3-letter codes stand for the island names: ANJ – Anjouan, MAY – Mayotte, MWA – Mohéli, GDC – Grand Comoro. Phylogenetic tree generated by the NJ method using a multiple sequence alignment.

Raw sequences were checked for sufficient length and quality. Sequences > 300 bp and with a quality > 70% were included in further analyses. They were then aligned with the MUSCLE algorithm (Edgar 2004) using default settings in the software Eugene. Individual sequence chromatograms were checked for ambiguous regions using the QIAGEN CLC Main Workbench software and low-quality regions were cut off manually. Cleaned sequences were aligned with reference sequences from BOLD and GenBank. A phylogenetic tree was calculated using the Neighbour-Joining method (NJ) via the IQ-TREE web server (Nguyen et al. 2015), with 1,000 bootstrap replicates to assess the reliability of the tree topology. Additionally, a Maximum Likelihood (ML) analysis was performed locally in IQ-TREE v.3 (Minh et al. 2020), using the best-fit substitution model GTR+F+I+G4 as selected by ModelFinder (Kalyaanamoorthy et al. 2017), with 1,000 ultrafast bootstrap replicates. The resulting ML tree was fully congruent with the Neighbour-Joining tree, confirming the robustness of the reported phylogenetic relationships (Fig. 2, Suppl. material 1).

## Data resources

All sequences are available in the BOLD Systems database under project code COMOR (Process IDS: COMOR001-26 to COMOR022-26).

## Checklists

### COMOR Orthoptera of the Comoros Archipelago

#### Oxya hyla

Serville, 1831

E3DEED3C-1023-5D4A-9786-807E504B450E

##### Materials

- **Type status:** Other material. **Occurrence:** occurrenceID: B2A0E5EA-643C-5D55-AE41-96588BA16C1A; **Taxon:** kingdom: Animalia; phylum: Arthropoda; class: Insecta; order: Orthoptera; family: Acrididae; genus: Oxya; specificEpithet: hyla; **Location:** continent: Africa; islandGroup: Comoros Archipelago; island: Anjouan; locality: Patsy, Site Universitaire de Patsy; verbatimElevation: 280 m; decimalLatitude: -12.1602; decimalLongitude: 44.4334; **Identification:** identifiedBy: Sylvain Hugel; dateIdentified: 24/11/2011; **Record Level:** institutionCode: University of Zurich (UZH)

#### Locusta migratoria

Linnaeus, 1758

3B6AA6A5-71DE-5932-BEA5-D07D22ECCE22

##### Materials

- **Type status:** Other material. **Occurrence:** occurrenceID: AD9A3BD5-DE09-5519-A75B-7BDCDE655F8F; **Taxon:** kingdom: Animalia; phylum: Arthropoda; class: Insecta; order: Orthoptera; family: Acrididae; genus: Locusta; specificEpithet: migratoria; **Location:** continent: Africa; islandGroup: Comoros; island: Grand Comoro; locality: Karthala, prairie; verbatimElevation: 1700 m; decimalLatitude: -11.7375; decimalLongitude: 43.3315; **Identification:** identifiedBy: Sylvain Hugel; dateIdentified: 26/04/2010; **Record Level:** institutionCode: University of Zurich (UZH)
- **Type status:** Other material. **Occurrence:** occurrenceID: 401D7F33-456B-5493-A782-C2D7E706E716; **Taxon:** kingdom: Animalia; phylum: Arthropoda; class: Insecta; order: Orthoptera; family: Acrididae; genus: Locusta; specificEpithet: migratoria; **Location:** continent: Africa; islandGroup: Comoros; island: Grand Comoro; locality: Karthala, prairie; verbatimElevation: 1700 m; decimalLatitude: -11.7375; decimalLongitude: 43.3315; **Identification:** identifiedBy: Sylvain Hugel; dateIdentified: 26/04/2010; **Record Level:** institutionCode: University of Zurich (UZH)

#### Afroxyrrhepes procera

(Burmeister, 1838)

F89AE939-714D-5D37-A88C-135655F5A054

##### Materials

- **Type status:** Other material. **Occurrence:** occurrenceID: D26F2BE4-0AB8-5E39-8311-ED0E1F6B71A0; **Taxon:** kingdom: Animalia; phylum: Arthropoda; class: Insecta; order: Orthoptera; family: Acrididae; genus: Afroxyrrhepes; specificEpithet: procera; **Location:** continent: Africa; islandGroup: Comoros; island: Mayotte; locality: Mamoudzou, Chissioua Mbouzi, savane; verbatimElevation: 44 m; decimalLatitude: -12.808715; decimalLongitude: 45.232373; **Identification:** identifiedBy: Sylvain Hugel; dateIdentified: 07/05/2024; **Record Level:** institutionCode: University of Zurich (UZH)
- **Type status:** Other material. **Occurrence:** occurrenceID: D5F5307B-A9E3-5C9E-B5A4-D0921B2F9EAA; **Taxon:** kingdom: Animalia; phylum: Arthropoda; class: Insecta; order: Orthoptera; family: Acrididae; genus: Afroxyrrhepes; specificEpithet: procera; **Location:** continent: Africa; islandGroup: Comoros; island: Mohéli; locality: Mlima Baoura; verbatimElevation: 294 m; decimalLatitude: -12.32146667; decimalLongitude: 43.68646667; **Identification:** identifiedBy: Sylvain Hugel; dateIdentified: 02/05/2010; **Record Level:** institutionCode: University of Zurich (UZH)

#### Acrotylus patruelis

(Herrich-Schäffer, 1838)

CCD97FFE-D65D-5163-BE03-114BB3F91365

##### Materials

- **Type status:** Other material. **Occurrence:** occurrenceID: 29436CD1-4F1B-54B6-8516-1EDE32D66BB3; **Taxon:** kingdom: Animalia; phylum: Arthropoda; class: Insecta; order: Orthoptera; family: Acrididae; genus: Acrotylus; specificEpithet: patruelis; **Location:** continent: Africa; islandGroup: Comoros; island: Grand Comoro; locality: Karthala, descente vers Krouani, chemin; verbatimElevation: 1150 m; decimalLatitude: -11.82; decimalLongitude: 43.4154; **Identification:** identifiedBy: Sylvain Hugel; dateIdentified: 01/12/2011; **Record Level:** institutionCode: University of Zurich (UZH)

#### Symbellia pallidafrons

Bruner, 1910

2C72CD7B-15EE-5FC3-8899-3CFEBC57B169

##### Materials

- **Type status:** Other material. **Occurrence:** occurrenceID: 8B8C0C8F-C4EE-5BCF-9042-C0D03A86C110; **Taxon:** kingdom: Animalia; phylum: Arthropoda; class: Insecta; order: Orthoptera; family: Euschmidtiidae; genus: Symbellia; specificEpithet: pallidafrons; **Location:** continent: Africa; islandGroup: Comoros; island: Grand Comoro; locality: Karthala, sentier Humblot; verbatimElevation: 869 m; decimalLatitude: -11.8359; decimalLongitude: 43.4038; **Identification:** identifiedBy: Sylvain Hugel; dateIdentified: 01/12/2011; **Record Level:** institutionCode: University of Zurich (UZH)
- **Type status:** Other material. **Occurrence:** occurrenceID: A1952E6E-0DCD-533D-92D8-EAE835E8BE45; **Taxon:** kingdom: Animalia; phylum: Arthropoda; class: Insecta; order: Orthoptera; family: Euschmidtiidae; genus: Symbellia; specificEpithet: pallidafrons; **Location:** continent: Africa; islandGroup: Comoros; island: Grand Comoro; locality: Nioumbadjou; verbatimElevation: 500 m; decimalLatitude: -11.8025; decimalLongitude: 43.3067; **Identification:** identifiedBy: Sylvain Hugel; dateIdentified: 18/08/2010; **Record Level:** institutionCode: University of Zurich (UZH)
- **Type status:** Other material. **Occurrence:** occurrenceID: 0E95AEBF-0895-5AA8-93EB-B708E9565078; **Taxon:** kingdom: Animalia; phylum: Arthropoda; class: Insecta; order: Orthoptera; family: Euschmidtiidae; genus: Symbellia; specificEpithet: pallidafrons; **Location:** continent: Africa; islandGroup: Comoros; island: Grand Comoro; locality: La Grille, sommet; verbatimElevation: 1050 m; decimalLatitude: -11.4726; decimalLongitude: 43.3457; **Identification:** identifiedBy: Sylvain Hugel; dateIdentified: 28/04/2010; **Record Level:** institutionCode: University of Zurich (UZH)

#### Gymnobothrus variabilis

Bruner, 1910

5A2894C5-CEF1-525C-AF89-55AA57C22F27

##### Materials

- **Type status:** Other material. **Occurrence:** occurrenceID: 493BB367-B27D-5BAD-8517-9E2AEDE5EA45; **Taxon:** kingdom: Animalia; phylum: Arthropoda; class: Insecta; order: Orthoptera; family: Acrididae; genus: Gymnobothrus; specificEpithet: variabilis; **Location:** continent: Africa; islandGroup: Comoros; island: Mohéli; locality: Mlima Baoura; verbatimElevation: 294 m; decimalLatitude: -12.32146667; decimalLongitude: 43.68646667; **Identification:** identifiedBy: Sylvain Hugel; dateIdentified: 02/05/2010; **Record Level:** institutionCode: University of Zurich (UZH)
- **Type status:** Other material. **Occurrence:** occurrenceID: B9B8ED39-F3FC-5942-9544-F1177B5C88A4; **Taxon:** kingdom: Animalia; phylum: Arthropoda; class: Insecta; order: Orthoptera; family: Acrididae; genus: Gymnobothrus; specificEpithet: variabilis; **Location:** continent: Africa; islandGroup: Comoros; island: Grand Comoro; locality: Batsa, littoral; verbatimElevation: 10 m; decimalLatitude: -11.6285; decimalLongitude: 43.2634; **Identification:** identifiedBy: Sylvain Hugel; dateIdentified: 30/04/2010; **Record Level:** institutionCode: University of Zurich (UZH)

#### Symbellia mayotteana

Descamps & Wintrebert, 1969

8DF86C68-0147-5B17-828F-B150BD360E0F

##### Materials

- **Type status:** Other material. **Occurrence:** occurrenceID: CCE271BD-5786-5914-8EFF-BD541E89CFE1; **Taxon:** kingdom: Animalia; phylum: Arthropoda; class: Insecta; order: Orthoptera; family: Euschmidtiidae; genus: Symbellia; specificEpithet: mayotteana; **Location:** continent: Africa; islandGroup: Comoros; island: Mayotte; locality: Tsingoni, Mlima Combani; verbatimElevation: 480 m; decimalLatitude: -12.804212; decimalLongitude: 45.152609; **Identification:** identifiedBy: Sylvain Hugel; dateIdentified: 02/05/2024; **Record Level:** institutionCode: University of Zurich (UZH)

#### Eyprepocnemis smaragdipes

Bruner, 1910

ACE5E130-F382-54DA-B837-3DD1299B8378

##### Materials

- **Type status:** Other material. **Occurrence:** occurrenceID: C0E35F63-5328-533A-A3EF-16866546DD90; **Taxon:** kingdom: Animalia; phylum: Arthropoda; class: Insecta; order: Orthoptera; family: Acrididae; genus: Eyprepocnemis; specificEpithet: smaragdipes; **Location:** continent: Africa; islandGroup: Comoros; island: Anjouan; locality: Patsy, Site Universitaire de Patsy; verbatimElevation: 280 m; decimalLatitude: -12.1602; decimalLongitude: 44.4334; **Identification:** identifiedBy: Sylvain Hugel; dateIdentified: 21/04/2010; **Record Level:** institutionCode: University of Zurich (UZH)

#### Atractomorpha acutipennis

(Guérin-Méneville, 1844)

72E457A7-590A-5BEE-B078-DCE667310691

##### Materials

- **Type status:** Other material. **Occurrence:** occurrenceID: A4087695-9529-56F6-8AD8-44E85A5CF223; **Taxon:** kingdom: Animalia; phylum: Arthropoda; class: Insecta; order: Orthoptera; family: Pyrgomorphidae; genus: Atractomorpha; specificEpithet: acutipennis; **Location:** continent: Africa; islandGroup: Comoros; island: Grand Comoro; locality: Tsije, jardin; verbatimElevation: 280 m; decimalLatitude: -11.7044; decimalLongitude: 43.2745; **Identification:** identifiedBy: Sylvain Hugel; dateIdentified: 29/04/2010; **Record Level:** institutionCode: University of Zurich (UZH)
- **Type status:** Other material. **Occurrence:** occurrenceID: A88CE61F-EF97-5A0D-9B20-3B26C8070689; **Taxon:** kingdom: Animalia; phylum: Arthropoda; class: Insecta; order: Orthoptera; family: Pyrgomorphidae; genus: Atractomorpha; specificEpithet: acutipennis; **Location:** continent: Africa; islandGroup: Comoros; island: Grand Comoro; locality: La Grille, pâture; verbatimElevation: 800 m; decimalLatitude: -11.4689; decimalLongitude: 43.3641; **Identification:** identifiedBy: Sylvain Hugel; dateIdentified: 23/08/2010; **Record Level:** institutionCode: University of Zurich (UZH)

#### Symbellia nigromaculata

Bruner, 1910

F770C356-E9D0-5CDB-B23B-96D1138FAFEF

##### Materials

- **Type status:** Other material. **Occurrence:** occurrenceID: A79D3EB2-6AA2-5F08-AFED-733389307AD4; **Taxon:** kingdom: Animalia; phylum: Arthropoda; class: Insecta; order: Orthoptera; family: Euschmidtiidae; genus: Symbellia; specificEpithet: nigromaculata; **Location:** continent: Africa; islandGroup: Comoros; island: Anjouan; locality: Patsy, Djadjana; verbatimElevation: 1046 m; decimalLatitude: -12.1817; decimalLongitude: 44.46156667; **Identification:** identifiedBy: Sylvain Hugel; dateIdentified: 24/11/2011; **Record Level:** institutionCode: University of Zurich (UZH)

#### Parepistaurus comoroensis

Descamps & Wintrebert, 1969

CF09B441-3275-5153-AFC7-D356AFFF3C6B

##### Materials

- **Type status:** Other material. **Occurrence:** occurrenceID: C82C29E1-EC81-5DEC-ABBA-001D757EFCA0; **Taxon:** kingdom: Animalia; phylum: Arthropoda; class: Insecta; order: Orthoptera; family: Acrididae; genus: Parepistaurus; specificEpithet: comoroensis; **Location:** continent: Africa; islandGroup: Comoros; island: Anjouan; locality: Patsy, Djadjana; verbatimElevation: 1046 m; decimalLatitude: -12.1817; decimalLongitude: 44.46156667; **Identification:** identifiedBy: Sylvain Hugel; dateIdentified: 24/11/2011; **Record Level:** institutionCode: University of Zurich (UZH)
- **Type status:** Other material. **Occurrence:** occurrenceID: 838903E4-2DC8-5818-BF59-7BF49086DC48; **Taxon:** kingdom: Animalia; phylum: Arthropoda; class: Insecta; order: Orthoptera; family: Acrididae; genus: Parepistaurus; specificEpithet: comoroensis; **Location:** continent: Africa; islandGroup: Comoros; island: Grand Comoro; locality: Karthala, descente vers Krouani, chemin; verbatimElevation: 1150 m; decimalLatitude: -11.82; decimalLongitude: 43.4154; **Identification:** identifiedBy: Sylvain Hugel; dateIdentified: 30/11/2011; **Record Level:** institutionCode: University of Zurich (UZH)
- **Type status:** Other material. **Occurrence:** occurrenceID: 80073B06-E0E6-50ED-92B7-B975AE3097A2; **Taxon:** kingdom: Animalia; phylum: Arthropoda; class: Insecta; order: Orthoptera; family: Acrididae; genus: Parepistaurus; specificEpithet: comoroensis; **Location:** continent: Africa; islandGroup: Comoros; island: Mohéli; locality: Mlima Baoura; verbatimElevation: 294 m; decimalLatitude: -12.3214667; decimalLongitude: 43.68646667; **Identification:** identifiedBy: Sylvain Hugel; dateIdentified: 04/12/2011; **Record Level:** institutionCode: University of Zurich (UZH)

#### Catantopsis sacalava

(Brancsik, 1893)

5D28A60A-5DC3-555E-B46F-6A67146E6747

##### Materials

- **Type status:** Other material. **Occurrence:** occurrenceID: 2866233D-2D6D-516F-A4CB-2962B0607963; **Taxon:** kingdom: Animalia; phylum: Arthropoda; class: Insecta; order: Orthoptera; family: Acrididae; genus: Catantopsis; specificEpithet: sacalava; **Location:** continent: Africa; islandGroup: Comoros; island: Mayotte; locality: Bandrele, Saziley Be; verbatimElevation: 44 m; decimalLatitude: -12.9781; decimalLongitude: 45.197536; **Identification:** identifiedBy: Sylvain Hugel; dateIdentified: 07/05/2010; **Record Level:** institutionCode: University of Zurich (UZH)

#### Aiolopus thalassinus

(Fabricius, 1781)

637DB454-9C05-5C28-8F0D-4C85BF941D39

##### Materials

- **Type status:** Other material. **Occurrence:** occurrenceID: EFFABFBE-8E5F-561D-B6C3-606835D20A37; **Taxon:** kingdom: Animalia; phylum: Arthropoda; class: Insecta; order: Orthoptera; family: Acrididae; genus: Aiolopus; specificEpithet: thalassinus; **Location:** continent: Africa; islandGroup: Comoros; island: Mayotte; locality: Tsingoni, Sohoa; verbatimElevation: 160 m; decimalLatitude: -12.804502; decimalLongitude: 45.1127; **Identification:** identifiedBy: Sylvain Hugel; dateIdentified: 07/05/2010; **Record Level:** institutionCode: University of Zurich (UZH)

#### Diabolocatantops axillaris

(Thunberg, 1815)

1B67E71D-0071-5A6A-B44A-34B8503AD224

##### Materials

- **Type status:** Other material. **Occurrence:** occurrenceID: 3B44BA0C-7549-57D4-8F47-C5E056302974; **Taxon:** kingdom: Animalia; phylum: Arthropoda; class: Insecta; order: Orthoptera; family: Acrididae; genus: Diabolocatantops; specificEpithet: axillaris; **Location:** continent: Africa; islandGroup: Comoros; island: Anjouan; locality: Papani, arrière de plage; verbatimElevation: 15 m; decimalLatitude: -12.804502; decimalLongitude: 44.5252; **Identification:** identifiedBy: Sylvain Hugel; dateIdentified: 16/08/2010; **Record Level:** institutionCode: University of Zurich (UZH)

## Analysis

The final dataset included 22 barcodes of 15 Caelifera species from 12 orthopteran genera (Fig. 2). One *Parepistaurus* sp. individual is a new record for Mohéli.

The bootstrap support for most clades was high (e.g. > 70%), indicating a high degree of confidence in the reliability of the clustering process. This suggests that the phylogenetic relationships within these genera are robust. Consequently, we propose the allocation of samples as presented in Table 1. The morphological identification of all specimens is congruent with the DNA sequences obtained by barcoding.

## Discussion

To date, published barcoding studies on the Comoros have focused on the following taxa: birds (Louette et al. 2011), reptiles (Hawlitschek et al. 2013, Hawlitschek et al. 2021) and parasites (Papa Mze et al. 2020). Grasshoppers in this region, however, have no published CO1 sequences or barcoding datasets yet, marking a clear gap. Grasshopper DNA barcoding has strong potential to contribute to both biodiversity research and food security in the Comoros. Outbreaks of orthoperan pest species, such as *Locusta migratoria*, pose a substantial threat to crop yields. On the other hand, given their high protein content, the diversity and use of edible orthopterans have been identified elsewhere, as, for example, in Madagascar (Van Itterbeeck et al. 2019, O’Hare et al. 2023). This can contribute to food web analyses by identifying grasshopper species, their host plants, predators and human users, allowing us to model trophic interactions and assessing how shifts in grasshopper species distributions or abundances under changing environmental or land-use conditions might affect food security (Van Itterbeeck et al. 2019). Mapping Comorian grasshopper diversity and their ecological roles via DNA barcoding could inform both conservation and agricultural management.

As described by Descamps & Wintrebert in 1969, 25 Comorian Acridomorpha species were discovered. Of the confirmed species, COI sequences of only two genera are found on GenBank: from *Oxya* sp. (*Oxya hyla*) and *Parepistaurus* sp. (*Parepistaurus comoroensis*). However, based on the sequences obtained in this study and the previous morphological identification, it appears that the number of species in the Comoros is higher.

In terms of biogeography, the records show rather disparate global distribution patterns. Species with a wide global distribution are *Locusta migratoria*, *Ailopus thalassinus*, *Diabolocatantops axillaris saucis* and *Acrotylus patruelis*, which are known to occur in parts of Europe, Asia and Africa. Mainly found on the African continent are *Afroxyrrhepes procera*, *Oxya hyla* and *Atractomorpha acutipennis*. In contrast, more localised species are *Gymnobothrus variabilis*, *Eyprepocnemis smaragdipes* and *Catantopsis sacalava*, which are only or mainly found in Madagascar. Finally, there are three endemic species, *Parepistaurus comoroensis*, *Symbellia mayotteana* and *Symbellia pallidafrons*, which are unique to the Comoros (Cigliano et al. 2024). The two *Symbellia* species have very similar COI sequences, raising the question of whether each island harbours a distinct subspecies or whether they should be considered conspecific.

We acknowledge that the present dataset, comprising 22 barcodes across 15 species constitutes a preliminary reference rather than a comprehensive barcode library. This limited sampling restricts the assessment of intraspecific variation and prevents a robust evaluation of the barcode gap for Comorian Caelifera. Nonetheless, our study represents the first molecular dataset for this group in the Archipelago and demonstrates the utility of DNA barcoding for poorly-studied taxa in island ecosystems. We therefore consider it a foundational resource that we hope will stimulate more comprehensive barcoding surveys of the invertebrate fauna of the Comoros Archipelago.

## Supplementary Material

XML Treatment for Oxya hyla

XML Treatment for Locusta migratoria

XML Treatment for Afroxyrrhepes procera

XML Treatment for Acrotylus patruelis

XML Treatment for Symbellia pallidafrons

XML Treatment for Gymnobothrus variabilis

XML Treatment for Symbellia mayotteana

XML Treatment for Eyprepocnemis smaragdipes

XML Treatment for Atractomorpha acutipennis

XML Treatment for Symbellia nigromaculata

XML Treatment for Parepistaurus comoroensis

XML Treatment for Catantopsis sacalava

XML Treatment for Aiolopus thalassinus

XML Treatment for Diabolocatantops axillaris

9C42F41A-5F76-573B-8CC8-F47FB2FC5A65 10.3897/BDJ.14.e188335.suppl1 Supplementary material 1 Phylogenetic relationships of orthopteran individuals on the Comoros Archipelago generated by the Maximum Likelihood method using a multiple sequence alignment Data type phylogenetic tree Brief description Samples of orthopteran individuals generated in this study are bold and clades belonging to the same subfamily are highlighted in different shades of the same colour. The 3-letter codes stand for the island names: ANJ – Anjouan, MAY – Mayotte, MWA – Mohéli, GDC – Grand Comoro. Phylogenetic tree generated by the Maximum Likelihood method using a multiple sequence alignment. File: oo_1586387.png https://binary.pensoft.net/file/1586387 Pauline Wasle, Oliver Hawlitschek, Sylvain Hugel

## Figures and Tables

**Table 1.** Taxonomic classification of sequenced grasshoppers sorted by island.

**Sample number**	**Island**	**Genus**	**Species**
PW 01	Anjouan	* Oxya *	* hyla *
PW 22	Anjouan	* Eyprepocnemis *	* smaragdipes *
PW 28	Anjouan	* Symbellia *	* nigromaculata *
PW 29	Anjouan	* Parepistaurus *	* comoroensis *
PW 42	Anjouan	* Diabolocatantops *	*axillaris saucius*
PW 09	Mohéli	* Afroxyrrhepes *	* procera *
PW 10	Mohéli	* Gymnobothrus *	* variabilis *
PW 32	Mohéli	* Parepistaurus *	* comoroensis *
PW 02	Grand Comoro	* Locusta *	* migratoria *
PW 04	Grand Comoro	* Acrotylus *	* patruelis *
PW 07	Grand Comoro	* Symbellia *	* pallidafrons *
PW 13	Grand Comoro	* Locusta *	* migratoria *
PW 21	Grand Comoro	* Symbellia *	* pallidafrons *
PW 26	Grand Comoro	* Symbellia *	* pallidafrons *
PW 27	Grand Comoro	* Gymnobothrus *	* variabilis *
PW 30	Grand Comoro	* Symbellia *	* pallidafrons *
PW 31	Grand Comoro	* Parepistaurus *	* comoroensis *
PW 39	Grand Comoro	* Atractomorpha *	* acutipennis *
PW 03	Mayotte	* Afroxyrrhepes *	* procera *
PW 18	Mayotte	* Symbellia *	* mayotteana *
PW 38	Mayotte	* Catantopsis *	* sacalava *
PW 40	Mayotte	* Aiolopus *	* thalassinus *
