# Supplementary figures and images for: First DNA barcode reference library of grasshoppers on the Comoros Archipelago
Source: Biodivers Data J. 2026 May 14;14:e188335. doi: 10.3897/BDJ.14.e188335 (PMC13195395; doi:10.3897/BDJ.14.e188335)

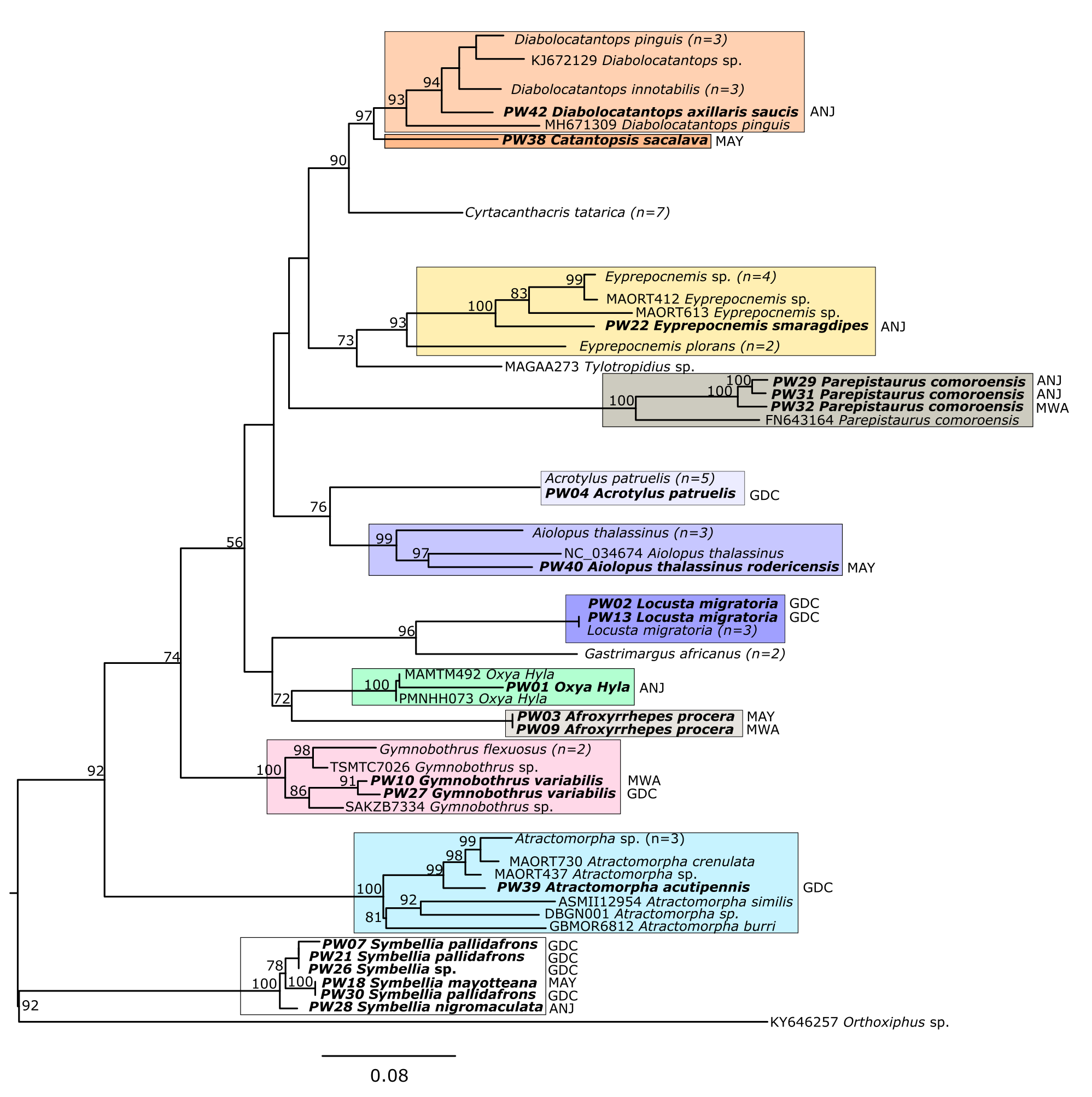

Supplement: Supplementary material 1 — Phylogenetic relationships of orthopteran individuals on the Comoros Archipelago generated by the Maximum Likelihood method using a multiple sequence alignment [file bdj-14-e188335-s001.png]
